# Technology-responsive regulation for genome editing: legal design for a rapidly evolving biotechnology

**DOI:** 10.3389/fbioe.2026.1837713

**Published:** 2026-06-17

**Authors:** Yong-Hong Bao, Agudamu, Yi Tu, Wei-Long Zhang, Peng-Fei Wei, Yang Zhang

**Affiliations:** 1 College of Physical Education, Hunan Normal University, Changsha, China; 2 Graduate School of Social Welfare, Sungkyunkwan University, Seoul, South Korea; 3 Independent Researcher, Windermere, FL, United States

**Keywords:** adaptive governance, bioethics, CRISPR-cas, genetic therapy, health policy

## Abstract

Genome editing has moved from an experimental research tool to a clinically relevant biotechnology, but its governance remains difficult because the technology itself continues to evolve. Existing debates have often focused on the ethical permissibility of specific applications, especially heritable human genome editing. This review argues that the broader challenge is one of regulatory design: legal and institutional frameworks must govern a field whose modalities, delivery systems, risks, and evidentiary standards are changing. Focusing on human biomedical genome editing, the article examines how scientific developments such as CRISPR-Cas9, base editing, and prime editing expose limitations in current international, national, and institutional governance arrangements. It then analyzes precautionary, innovation-permissive, risk-based, responsive, adaptive, and anticipatory approaches, arguing that each captures part of the problem but remains incomplete when the object of regulation is dynamic. To address this gap, the article proposes technology-responsive regulation (TRR) as a legal-regulatory framework built around adaptive regulatory capacity, scientific-institutional integration, and multilevel coordination. TRR organizes familiar governance tools, including periodic review, differentiated categorization, scientific advice, registries, public engagement, enforcement, and international coordination, around the task of maintaining accountable oversight as genome editing changes. Responsible governance requires not only stronger rules, but institutions capable of revising them without losing legality, accountability, or public trust.

## 1 Introduction

Genome editing has become a central technology in contemporary biotechnology because it changes not only what can be done to biological systems, but also how quickly such interventions can move from laboratory research toward clinical and commercial use. Earlier genetic engineering techniques were often technically demanding, costly, and relatively inflexible. By contrast, clustered regularly interspaced short palindromic repeats (CRISPR)-based systems made targeted genomic intervention more programmable, accessible, and scalable. The demonstration that CRISPR-associated protein 9 (Cas 9) can be guided by RNA to introduce sequence-specific DNA cleavage established a platform of major scientific importance (Jinek et al., 2012), and subsequent work in mammalian cells confirmed its relevance for biomedical research and translation (Cong et al., 2013; Mali et al., 2013).

The biomedical promise of genome editing is substantial. It has enabled advances in functional genomics, disease modeling, therapeutic development, and potentially curative interventions for severe genetic disease. Clinical applications for sickle cell disease and β-thalassemia show that genome editing is no longer merely a speculative technology, but part of an emerging therapeutic landscape (Frangoul et al., 2021). At the same time, the field continues to change. Base editing and prime editing have expanded the range of possible interventions and altered the technical logic of editing by reducing or avoiding some forms of double-strand-break-dependent repair (Xu et al., 2024). These developments complicate regulatory assessment because the relevant technology is not fixed. Genome editing is better understood as an evolving family of tools, delivery systems, applications, and risk profiles.

This technological dynamism has direct legal significance. Regulatory systems depend on categories, standards, and institutional procedures that are sufficiently stable to support accountability and consistent decision-making. Genome editing unsettles those conditions. A regulatory framework developed around one generation of editing tools may become incomplete when new modalities, delivery systems, or clinical contexts emerge. Law is therefore not only asked to decide whether particular applications should be permitted or prohibited. It is also asked to govern a field in which the object of regulation continues to change.

The permissibility of heritable human genome editing has understandably occupied a central place in legal and ethical debate. Because germline interventions may introduce changes affecting future persons who cannot consent, they raise questions concerning human dignity, reproductive liberty, disability justice, intergenerational responsibility, and the social meaning of genetic intervention (Almeida et al., 2022; Martani et al., 2025). Yet a governance analysis limited to germline permissibility risks missing a wider problem. Genome editing is not only ethically controversial; it is technically and institutionally difficult to govern because its modalities, evidentiary demands, and risk profiles continue to evolve.

The 2018 “CRISPR twin” incident made this difficulty visible on a global scale. The reported editing and implantation of human embryos, resulting in the birth of genetically modified children, was widely condemned as scientifically premature and ethically indefensible (Greely, 2019). The episode also exposed weaknesses in institutional review, professional self-regulation, domestic oversight, and international coordination (Chen et al., 2022). Still, the case should not be treated as the only or even the paradigmatic governance problem in genome editing. It involved a dramatic violation of accepted scientific and ethical standards, but many regulatory challenges are less visible: how to update safety assessment as editing tools change; how to distinguish research, clinical innovation, and therapy; how to coordinate domestic law with international soft-law guidance; how to oversee emerging modalities such as base editing; and how to include public and stakeholder perspectives without reducing engagement to a symbolic exercise.

Genome editing therefore belongs to a broader class of rapidly evolving technologies that challenge traditional regulatory design. Similar concerns have been observed in artificial intelligence, synthetic biology, neurotechnology, and data-intensive health systems, where legal systems confront the pacing problem: the tendency of technological development to move faster than the legal and ethical structures designed to oversee it (Marchant et al., 2011; Wang et al., 2023). In the genome editing context, however, the pacing problem has distinctive force. The technology implicates bodily integrity, reproduction, inheritance, and human identity, while the scientific basis for assessing risk remains unsettled. Law is not merely lagging behind established knowledge; it is often regulating while the relevant knowledge is still being produced.

Existing governance approaches provide important resources but remain incomplete when considered alone. Precautionary approaches emphasize restraint under uncertainty, especially where irreversible harm may occur. Risk-based regulation helps differentiate oversight according to probability, magnitude, and context of harm. Innovation-permissive approaches seek to avoid unnecessary barriers to therapeutic development. Adaptive and anticipatory governance frameworks highlight learning, revision, and early reflection (Nelson et al., 2021; Barlevy et al., 2024; Mourby et al., 2022). Each contributes something valuable. The difficulty is that genome editing requires a framework that can combine scientific updating with legal accountability. Flexibility alone is not sufficient; nor is prohibition alone. A credible legal response must preserve rule-of-law commitments while enabling institutions to revise classifications, standards, and oversight practices as the technology develops.

Much of the international discussion of genome editing has been conducted through soft-law reports, ethics commissions, expert bodies, and professional organizations (Andorno et al., 2020; Chan et al., 2015; Ormond et al., 2017). These efforts have produced valuable principles, including transparency, caution, inclusiveness, social justice, and public engagement. Yet principles alone do not resolve the institutional question of how governance should be structured when the object of governance is changing. A legal system may endorse caution while lacking mechanisms for dynamic review. It may prohibit one class of interventions while failing to classify the next-generation of technologies coherently. It may invoke ethical oversight while under-specifying the relationship between scientific expertise, administrative authority, and enforceable legal standards.

Genome editing is a particularly revealing site for this problem because it combines therapeutic promise with unusually high normative stakes. The same field that offers prospective treatment for severe genetic disease also raises concerns about irreversible harm, reproductive misuse, unequal access, and the social meaning of human genetic intervention. The central challenge is therefore not only to decide what genome editing law should permit or prohibit, but to design institutions capable of revising, differentiating, and justifying regulatory judgments as the technology changes.

Accordingly, the analysis below develops a structural legal-regulatory model for a biotechnology whose scientific evolution is constitutive of the governance challenge itself. The article concentrates on human biomedical genome editing, where editing technologies intersect with clinical risk, bodily integrity, reproductive decision-making, embryo research, and possible intergenerational effects.

## 2 Scientific evolution of genome editing technologies

The regulatory significance of genome editing cannot be understood without attention to the pace and direction of its scientific development. Law does not govern a single stable technique called “genome editing.” It governs an evolving family of tools whose mechanisms, applications, delivery systems, and risk profiles differ in legally relevant ways. When those features change, regulatory assumptions about risk, evidence, and institutional competence may also need to be reconsidered.

CRISPR-Cas9 became foundational because it combined programmability, efficiency, and relative accessibility. Earlier genome editing systems, including zinc-finger nucleases and TALENs, had shown that targeted genomic modification was possible, but CRISPR-Cas9 simplified experimental design by using guide RNA to direct a nuclease to a genomic target (Jinek et al., 2012). Its adaptation to mammalian and human cells expanded the practical reach of genome modification (Cong et al., 2013; Mali et al., 2013). This shift also had regulatory consequences: a technology that is easier to design, cheaper to use, and more readily disseminated creates more sites of potential intervention across research, clinical, and commercial settings.

The original CRISPR-Cas 9 system operates by producing double-strand breaks at specified genomic loci. Those breaks are repaired by endogenous cellular pathways, particularly non-homologous end joining and homology-directed repair. These mechanisms can be used to disrupt genes, introduce targeted mutations, or insert donor sequences (Jinek et al., 2012). From a regulatory perspective, this mechanism created a distinctive safety problem. The intended edit was only one part of the relevant assessment. Regulators and ethics committees also had to consider off-target effects, variable repair outcomes, mosaicism, unintended insertions or deletions, and broader consequences of DNA break repair.

These concerns have been reinforced by subsequent evidence. Early work showed that CRISPR-Cas nucleases could generate off-target mutations in human cells (Fu et al., 2013). Later studies raised additional concerns about large deletions, chromosomal rearrangements, and other structural changes associated with double-strand-break repair (Aussel et al., 2025; Cullot et al., 2025; Kosicki et al., 2018). The legal relevance of these findings is that safety assessment cannot be reduced to confirming whether the intended sequence change occurred. A regulatory framework also needs to ask whether the methods used to detect unintended effects are adequate, whether downstream biological consequences have been sufficiently characterized, and whether long-term monitoring is required after clinical use.

Scientific development has responded to some of these concerns by producing editing systems that modify DNA without relying in the same way on double-strand breaks. Base editing is the most important example for regulatory analysis. Cytosine and adenine base editors enable direct conversion of one nucleotide base into another at targeted genomic sites, thereby allowing some pathogenic point mutations to be corrected without conventional double-strand-break-dependent editing (Gaudelli et al., 2017; Komor et al., 2016). Prime editing extends this development by enabling targeted substitutions, insertions, and deletions without the same reliance on donor-template repair or blunt double-strand-break induction (Anzalone et al., 2019).

Base editing illustrates why technological variation changes governance requirements rather than merely adding scientific detail. At first glance, a system that avoids double-strand breaks may appear easier to regulate because it may reduce some structural risks associated with nuclease-based editing. But the regulatory question does not disappear; it changes form. Base editing raises concerns about bystander edits within the editing window, off-target deamination in DNA or RNA, editor persistence, delivery-related toxicity, and the adequacy of assays used to detect unintended base conversions. In hematopoietic stem cells, for example, emerging evidence suggests that base and prime editing may have genotoxic effects that require careful assessment rather than a presumption of safety based on mechanistic precision alone (Fiumara et al., 2024).

This example has direct implications for regulatory design. A framework developed mainly around double-strand-break risks may ask the wrong questions when applied to base editing. It may focus heavily on large deletions and repair outcomes while giving insufficient attention to bystander editing, deaminase activity, tissue-specific effects, or transcriptomic consequences. Conversely, a framework that treats base editing as simply “safer CRISPR” may underestimate uncertainty because it equates reduced reliance on double-strand breaks with overall risk reduction. A TRR system would not classify base editing only by analogy to earlier CRISPR-Cas9 systems. It would require updated evidentiary standards, modality-specific safety endpoints, and review procedures capable of revising guidance as new data emerge.

The same point applies to legal categorization. Regulatory systems often distinguish between somatic and germline editing, research and therapy, laboratory study and clinical intervention, and product and procedure. These distinctions remain important, but they do not fully capture the regulatory significance of technological variation. A base-editing intervention used *ex vivo* in hematopoietic stem cells differs from an *in vivo* editing intervention delivered systemically through viral vectors or lipid nanoparticles. A prime-editing platform may raise different questions again, depending on the target tissue, delivery method, persistence of editing components, and expected durability of effect. In each case, the governance issue is not only whether genome editing should be permitted, but what kind of evidence and oversight the specific modality requires.

Clinical translation intensifies these concerns. *Ex vivo* editing of hematopoietic stem cells has already shown therapeutic potential for blood disorders, while *in vivo* approaches are expanding the range of possible targets through viral and non-viral delivery systems (Kazemian et al., 2022; Lee et al., 2025; Johnson et al., 2024). Once genome editing enters clinical pathways, regulatory assessment must address patient selection, informed consent, manufacturing quality, trial design, post-treatment monitoring, long-term follow-up, and responsibility for delayed adverse effects. These requirements are familiar in biomedical regulation, but genome editing complicates them because the relevant risks may change as editing platforms evolve.

The scientific uncertainty surrounding genome editing is therefore recurrent rather than temporary. New tools may reduce familiar risks while introducing different ones; new delivery systems may make previously speculative applications clinically plausible; and new evidence may alter the safety assumptions on which earlier regulatory positions were based. The legal significance of technological evolution is therefore twofold. It changes what must be assessed by altering the mechanisms, risks, and applications of genome editing, and it changes the form of governance required because oversight systems designed for a relatively stable technology may become misaligned with a field that continues to diversify.

## 3 Existing genome editing governance frameworks

Genome editing is not governed through a single coherent legal regime. It is regulated through a layered combination of international soft law, national legislation, administrative regulation, professional standards, institutional ethics review, clinical-trial oversight, and funding restrictions. This structure is not unusual in biotechnology governance, but it is particularly important for genome editing because the field is transnational, technically dynamic, and normatively contested. The problem is therefore not the absence of governance. It is the uneven fit between existing governance arrangements and a technology whose forms, applications, and risks continue to evolve.

At the international level, genome editing has been governed mainly through soft-law instruments. There is no comprehensive global treaty devoted specifically to genome editing. Instead, international governance has developed through summit statements, expert reports, ethics recommendations, and guidance issued by scientific academies, bioethics bodies, and intergovernmental organizations. Soft law has advantages in this field: it can be produced more quickly than treaties, accommodate scientific uncertainty, and support transnational norm formation where formal agreement may be difficult (Qin et al., 2026; Abbott and Snidal, 2000). Its weakness is that it depends on domestic uptake and institutional implementation. It can articulate norms but cannot by itself ensure enforceable coordination.

The 2015 International Summit on Human Gene Editing was an early example of this approach. It treated clinical germline editing as irresponsible unless safety and efficacy concerns had been addressed and broader societal consensus had been reached (Baltimore et al., 2015; LaBarbera, 2016). The summit did not create binding law, but it helped establish a transnational baseline of caution and scientific responsibility. Subsequent reports adopted a more differentiated position. The United States National Academies report suggested that clinical germline editing might, in principle, be considered under highly restricted conditions, especially for preventing serious disease where no reasonable alternatives exist (National Academies of Sciences, Engineering, and Medicine, National Academy of Medicine, National Academy of Sciences, Committee, 2017). The Nuffield Council on Bioethics similarly concluded that heritable genome editing could be ethically acceptable if consistent with the welfare of the future person and with principles of social justice and solidarity (Nuffield Council on Bioethics, 2018). These reports show that international governance has not been limited to prohibition. It has instead moved between restraint, conditional permissibility, and procedural safeguards.

The World Health Organization’s 2021 framework and recommendations attempted to make this discussion more institutionally practical. They emphasized transparency, registry-based governance, ethical oversight, public engagement, and international cooperation (World Health Organization, 2021a; World Health Organization, 2021b). This was significant because it framed genome editing governance not only as a matter of moral principle, but also as a problem of governance infrastructure. Even so, the WHO recommendations remain non-binding. They can guide states and institutions, but they cannot ensure consistent implementation across jurisdictions.

National legal frameworks therefore carry most of the practical burden. In the United States, governance is fragmented across administrative regulation, funding rules, institutional review, and statutory restrictions. Somatic genome editing therapies fall mainly within the regulatory authority of the Food and Drug Administration (FDA), which assesses safety, efficacy, manufacturing, clinical-trial design, and long-term follow-up for gene therapy products (Eisenman and Swindle, 2022). This allows some flexibility for therapeutic innovation. By contrast, reproductive germline editing is restricted through a more indirect legal structure. Federal appropriations riders prevent the FDA from considering applications involving genetically modified embryos intended for pregnancy, while federal funding restrictions also limit certain embryo research pathways (Matthews and Morali, 2022). The result is a strong practical barrier, but one built through piecemeal mechanisms rather than a single integrated genome-editing statute.

European governance is more explicitly shaped by human dignity, rights protection, and precaution. The Council of Europe’s Oviedo Convention prohibits interventions seeking to modify the human genome in ways that would be inherited by descendants and remains an important reference point in European biolaw (Nawrot, 2018). Several European jurisdictions restrict germline editing while allowing embryo research only under defined conditions (Baylis et al., 2020; Nordberg et al., 2020). Yet even this framework is not without interpretive difficulty. Scholars have debated whether the Oviedo Convention categorically prohibits all forms of germline editing in every relevant sense (Beriain et al., 2019). More broadly, European governance illustrates both the strength and the limits of principled constraint. Strong ex ante limits can protect against premature or ethically unacceptable applications, but they may also struggle to account for technological differentiation across editing modalities, therapeutic contexts, and research uses.

China provides a different example. Before the CRISPR twin incident, governance of human embryo editing in China was often criticized as formally present but institutionally weak in enforcement and coordination. The post-2018 response included legal and administrative reforms, stronger ethical review requirements, clearer institutional responsibilities, and increased penalties for unauthorized experimentation involving human embryos or human genetic technologies (Chen et al., 2022; Zou et al., 2025). The 2024 Ethical Guidelines for Human Genome Editing Research further emphasized institutional ethics review, risk assessment, and the prohibition of reproductive use involving edited embryos intended for implantation (Ministry of Science and Technology of the People’s Republic of China, 2024). These reforms strengthened formal oversight, but they also illustrate the reactive pattern common in emerging-technology governance: institutional redesign often follows scandal rather than anticipating the conditions that make governance failure possible.

Across jurisdictions, several common features emerge. Most systems distinguish between somatic and germline interventions, treating germline or reproductive uses as more sensitive because of their heritable implications. Many systems rely on inherited legal categories, including gene therapy regulation, embryo research law, human-subjects review, product regulation, and professional ethics. International guidance remains largely soft-law based. Institutional ethics review plays a central role, but often within legal frameworks that are incomplete, unevenly specified, or technologically lagging.

These features should not be dismissed. Existing governance arrangements have created meaningful constraints and supported legitimate therapeutic development in some contexts. Yet they remain patchwork arrangements shaped by historical layering rather than by deliberate design for a rapidly evolving technology. As genome editing diversifies into base editing, prime editing, epigenome editing, improved delivery systems, and increasingly plausible *in vivo* applications, the central question is not whether genome editing is regulated. It is whether existing regulation is sufficiently responsive to scientific change while remaining legally accountable.

## 4 Structural limits of existing legal governance

The governance difficulties associated with genome editing are not reducible to disagreement over particular applications. They arise from a deeper structural problem: legal systems are being asked to govern a technological field whose capabilities, applications, and risk profiles evolve more quickly than the frameworks created to regulate them. Regulatory scholarship often describes this as the pacing problem: the mismatch between technological development and the slower tempo of legal and ethical oversight (Marchant et al., 2011; Wang et al., 2023). In genome editing, however, the issue is not only speed. It is also legal fit.

If regulatory lag were simply a matter of delay, the solution would be to legislate faster, revise guidance more frequently, or accelerate administrative review. Genome editing presents a more difficult problem. CRISPR-Cas9, base editing, prime editing, epigenome editing, multiplex editing, and new delivery platforms do not raise identical legal and safety questions (Alex and Winkler, 2024; Makarova et al., 2025; Kim et al., 2025). They differ in mechanism, error profile, delivery risk, therapeutic plausibility, and evidentiary requirements. What lags, therefore, is often the conceptual fit between inherited legal categories and the changing scientific object being regulated.

Law commonly manages new technologies by placing them within existing categories. This strategy can promote stability and predictability. New biological products can be assessed under biologics frameworks; clinical research can be reviewed through human-subjects protections; embryo research can be governed through existing reproductive or developmental biology rules. But this approach works only when inherited categories remain descriptively and normatively adequate. Genome editing strains that assumption. Categories such as somatic versus germline, therapy versus enhancement, research versus clinical use, and product versus procedure remain important, but they may not be fine-grained enough to capture differences among editing modalities, delivery methods, and clinical contexts.

This creates two risks. The first is under-inclusion: new developments may escape meaningful scrutiny because they do not fall clearly within existing legal concepts. The second is over-generalization: legal rules may treat heterogeneous technologies as though they presented the same risks simply because they share the label “genome editing.” Both risks undermine legitimacy. Under-inclusion leaves gaps in protection, while over-generalization can produce rules that are scientifically imprecise and normatively overbroad.

Scientific uncertainty compounds this problem. Regulatory institutions do not govern genome editing against a background of settled knowledge. They govern while scientific understanding of the technology’s risks is changing. Initial concern centered on off-target effects and unintended sequence changes. Later work broadened the risk horizon to include large deletions, chromosomal rearrangements, p53-mediated DNA damage responses, bystander edits, inflammatory effects, cellular senescence, and other consequences that may not be captured by narrow genotyping endpoints (Bantele et al., 2025; Conti et al., 2025; Haapaniemi et al., 2018; Kalter et al., 2025; Zhu et al., 2025). These findings do not simply add more items to a safety checklist. They may change what counts as adequate evidence of safety.

This is especially important because uncertainty in genome editing is recurrent rather than temporary. A new editing modality may reduce one class of risks while introducing another. A new delivery system may make a previously speculative application clinically plausible. A new assay may reveal risks that earlier methods missed. Governance therefore cannot rely entirely on one-time authorization decisions. It must connect authorization, evidence generation, monitoring, and revision.

Institutional constraints also matter. Legislatures are not designed for constant technical recalibration. Administrative agencies can update guidance more readily, but they remain constrained by statutory authority, procedure, resources, and judicial review. Ethics committees can assess protocol-level risks, but they may lack the mandate or consistency required for system-wide governance. Expert advisory bodies can interpret scientific change, but their role must be linked to accountable public authority. These are not simply institutional defects. They are part of the way legal legitimacy is secured. But they create friction when the regulated field changes rapidly.

Genome editing further complicates governance because it is transnational. Research collaborations, clinical development, investment, and scientific publication move across borders more easily than legal authority does. A restrictive jurisdiction may reduce domestic risk but cannot fully prevent activity from shifting elsewhere. International soft law can support coordination, but it lacks the enforcement capacity of national law. This creates persistent opportunities for uneven implementation and regulatory arbitrage (Abbott and Snidal, 2000; Baylis et al., 2020; Townsend, 2020; Yu et al., 2021).

The result is a governance landscape that is neither empty nor fully coherent. Existing models each capture part of the problem. Precautionary governance protects against irreversible harm, especially in heritable contexts. Risk-based regulation supports proportionality. Innovation-permissive approaches protect therapeutic development from unnecessary obstruction. Adaptive and anticipatory governance emphasize learning and early reflection. Responsive regulation contributes ideas about calibration and graduated enforcement. Yet none of these approaches, standing alone, fully addresses the legal problem created by a technology whose object, risks, and evidentiary requirements continue to change.

What is missing is a framework that treats technological evolution as a first-order legal problem. Genome editing governance should not only ask whether a given application is safe enough, ethical enough, or socially acceptable enough. It must also ask whether the regulatory system is capable of revising its categories, standards, and institutional relations as the science changes. This is the problem to which TRR is directed.

## 5 Technology-responsive regulation: toward a legal theory of disciplined governance

Technology-responsive regulation (TRR) is proposed here as a legal-regulatory framework for domains in which technological capabilities, applications, and risk profiles evolve rapidly enough to threaten the adequacy of static governance. The concept is not intended to suggest that law should simply follow science or defer to technical expertise. Nor does it imply that flexibility is always preferable to constraint. Its central claim is narrower: where the object of regulation is itself changing, legal systems need structured mechanisms for updating classifications, evidentiary standards, and oversight practices without abandoning legality, accountability, transparency, and rights protection. TRR differs from a general call for flexible governance. Flexibility can become discretionary, opaque, or technocratic if it is not organized through law. A technology-responsive framework requires identifiable authority, public reasons, procedural safeguards, and reviewable decisions. It also accepts that some applications may remain subject to strong ex ante limits. Responsiveness does not mean that every boundary is negotiable. It means that the surrounding governance system must be capable of learning and revising where scientific change alters the assumptions on which regulation depends.

### 5.1 Locating TRR within regulatory theory

TRR draws on, but is not identical to, several established strands of regulatory theory. Responsive regulation is especially useful because it rejects rigid oppositions between command-and-control and deregulation. It shows that oversight can be calibrated to context and conduct (Ayres and Braithwaite, 1992). Genome editing, however, requires responsiveness not only to the behavior of regulated actors, but also to the changing features of the technology itself. A compliant investigator using a new editing modality may still present regulatory questions that older categories do not capture.

Risk-based regulation (Hood et al., 2001) also contributes to TRR because genome editing should not be governed as a single undifferentiated category. Somatic *ex vivo* editing for severe disease, *in vivo* systemic editing, embryo research, and reproductive germline intervention do not present the same legal or ethical profile. A proportional system should distinguish among them. Yet risk-based regulation reaches its limits when the relevant evidence changes. TRR therefore treats risk calibration as a continuing institutional task rather than a one-time classification exercise.

Adaptive and anticipatory governance provide a further foundation. They emphasize learning, foresight, upstream reflection, and revision in response to new information (Nelson et al., 2021; Barlevy et al., 2024; Mourby et al., 2022; Rueda et al., 2025; van der Weij et al., 2026). These ideas are important, but they often remain expressed at a high level of institutional aspiration. TRR gives them a more explicitly legal form by asking who has authority to revise standards, what procedures should govern revision, how expert input should be incorporated, and how revised standards become publicly accountable. The relationship between TRR and adjacent governance models is summarized in Table 1.

**TABLE 1:** Governance approaches and the contribution of technology-responsive regulation (TRR).

Governance approach	Useful contribution	Limitation when technology evolves	How TRR incorporates or revises it
Precautionary governance	Emphasizes restraint under uncertainty, especially where irreversible or heritable harm is possible	Can become static or overbroad if it does not distinguish among technologies, uses, and changing evidence	Preserves bounded precaution while requiring review mechanisms when scientific developments alter the basis for regulatory judgment
Innovation-permissive governance	Supports therapeutic development and avoids unnecessary barriers to biomedical innovation	May overestimate the capacity of existing biomedical pathways to absorb new editing modalities	Allows innovation but asks whether inherited regulatory pathways remain fit for the specific technology and use
Risk-based regulation	Calibrates oversight according to probability, magnitude, and context of harm	Depends on risk categories and evidence that may become outdated as editing tools and delivery systems change	Treats risk assessment as dynamic, modality-specific, and revisable
Responsive regulation	Provides graduated oversight and enforcement calibrated to conduct and compliance behavior	Responds mainly to regulated actors rather than to changes in the technology itself	Extends responsiveness from actor behavior to technological evolution and regulatory fit
Adaptive and anticipatory governance	Emphasizes learning, foresight, early reflection, and iterative revision	Often under-specifies legal authority, enforceability, and accountability	Embeds adaptive learning within legal procedures, public reasons, and accountable institutions
Technology-responsive regulation	Integrates scientific updating, legal accountability, institutional learning, and multilevel coordination	Institutionally demanding and requires clear allocation of authority	Provides a legal framework for governing technologies whose risks, applications, and evidentiary requirements continue to change

### 5.2 TRR: definition, normative justification, and core pillars

TRR can be defined as a legal-regulatory framework designed for domains in which technological capabilities, applications, and risk profiles evolve rapidly enough to threaten the adequacy of static governance. Three features distinguish TRR. First, it treats technological evolution itself as legally relevant. The governance problem is not only that actors may misuse genome editing, but that the technology’s mechanisms, risk profile, and applications may change in ways that alter the adequacy of existing law. Second, it requires institutionalized revision. Regulatory updating should not depend only on scandal, informal expert consensus, or *ad hoc* administrative adjustment. There must be structured pathways for monitoring scientific developments, reconsidering classifications, revising guidance, and giving reasons for change. Third, it remains bounded by legal constraint. Adaptation must occur through transparent, accountable, and reviewable procedures.

The normative justification for TRR rests on legitimacy, accountability, and precaution. Regulation is more legitimate when its categories and standards remain connected to the realities they govern. In fast-moving technological fields, rules designed for earlier conditions may become either under-protective or overbroad. Adaptive capacity can therefore support, rather than weaken, legitimacy. Accountability also improves when adaptation is brought into visible legal form. Without formal revision pathways, governance may shift into informal professional norms, unpublished agency interpretations, or expert advice whose legal status is unclear (Black, 2008; Marchant, 2021). TRR seeks to make such adaptation explicit and institutionally accountable. Precaution is also better understood dynamically. Precaution does not require regulatory immobility (Kaebnick et al., 2016). In genome editing, genuine caution may require updating standards when new evidence changes what counts as risk, uncertainty, or adequate monitoring. A legal regime that refuses to update in light of new safety evidence is not necessarily precautionary; it may simply be obsolete. TRR therefore treats precaution and adaptation as compatible. Some uses may remain prohibited or strongly restricted, while other areas require ongoing reassessment as technologies and evidence develop.

For genome editing governance, TRR rests on three core pillars. The first is adaptive regulatory capacity: the ability of legal institutions to revise classifications, evidentiary requirements, and oversight practices in response to significant scientific developments. This may involve statutory review clauses, delegated technical rulemaking, mandatory reassessment triggers, or periodic guidance revision. The important point is that revision becomes a normal feature of governance rather than an exceptional response to crisis.

The second pillar is scientific-institutional integration. Genome editing governance cannot function without scientific expertise, but expertise must be incorporated into public authority in a legally intelligible way. Expert advisory bodies, technical review committees, and interdisciplinary panels should help identify which developments are regulatory significant. Yet their recommendations should be linked to transparent decision-making by agencies, legislatures, or other public bodies. Expertise should inform legal authority, not replace it.

The third pillar is multilevel coordination. Genome editing develops through international research networks, commercial pipelines, clinical collaborations, journals, funders, and professional bodies. National law remains the principal source of enforceable authority, but it cannot govern the field alone. TRR therefore requires coordination among domestic law, international soft law, scientific standards, registries, reporting systems, and cross-border regulatory learning. Such coordination does not require global uniformity. It requires enough alignment to reduce avoidable gaps, improve transparency, and limit opportunities for regulatory arbitrage.

TRR is therefore best understood as a legal theory of disciplined adaptability within principled limits. Its contribution is not the invention of every instrument it uses. Advisory committees, risk classification, public engagement, registries, review clauses, and enforcement mechanisms already exist in different forms. The contribution lies in organizing these instruments around a specific problem: how law can govern a technology whose scientific evolution is part of the governance challenge itself.

## 6 Institutional architecture for technology-responsive governance of genome editing

A legal theory becomes meaningful only when translated into institutional design. TRR does not require a single global model, since legal systems differ in constitutional structure, administrative capacity, political culture, and ethical commitments. Its purpose is instead to identify the institutional functions needed to govern genome editing under conditions of scientific change. Under TRR, genome editing governance should not depend on a single institution. Legislatures, regulatory agencies, ethics committees, scientific advisory bodies, courts, professional organizations, funders, journals, public engagement mechanisms, and international organizations each perform different functions. Table 2 summarizes this architecture.

**TABLE 2:** Institutional architecture for genome editing governance under technology-responsive regulation (TRR).

Governance actor	Primary governance role	TRR instrument	Intended function
Legislature	Establish high-level legal boundaries and authorize adaptive review mechanisms	Statutory prohibitions, review clauses, delegated rulemaking authority	Preserve democratic legitimacy while enabling structured regulatory updating
Regulatory agencies	Oversee therapeutic translation and revise technical oversight standards	Periodic guidance revision, tiered authorization, post-trial and long-term follow-up requirements	Improve legal fit between evolving science and regulatory practice
Specialized high-risk review body	Evaluate boundary-crossing or high-consequence genome editing proposals	Central referral, enhanced approval, cross-agency consultation	Ensure consistent oversight of reproductive, embryo-related, and first-in-human high-risk uses
Institutional ethics committees	Conduct protocol-level review of research and clinical proposals	Genome-editing-specific review criteria, escalation triggers, compliance monitoring	Strengthen local oversight while preventing ethics review from substituting for system-level regulation
Scientific advisory structures	Translate scientific developments into governance-relevant assessments	Public expert reports, risk horizon scanning, evidentiary recommendations	Integrate evolving scientific knowledge into accountable legal authority
Digital regulatory infrastructures	Support visibility, coordination, and ongoing monitoring	Registries, reporting systems, horizon-scanning platforms, audit tools	Improve transparency, timeliness, and cross-institutional regulatory awareness
International organizations and coordination networks	Facilitate cross-border information exchange and normative alignment	Shared registries, reporting standards, coordination forums, soft-law guidance	Reduce regulatory arbitrage and strengthen procedural harmonization
Public and stakeholder engagement mechanisms	Supply social legitimacy and structured public justification	Consultation, notice-and-comment, deliberative forums, stakeholder review channels	Ensure genome editing governance remains publicly accountable and normatively grounded

### 6.1 Dynamic regulatory review as a standing institutional function

The first institutional implication of TRR is that review of genome editing regulation should become a standing function rather than an exceptional response to scandal. Many legal systems revisit biotechnology law only after controversy exposes a gap. This reactive pattern is understandable, but it is poorly suited to a field in which scientific change may become regulatory significant before it becomes publicly visible.

A TRR framework should therefore include formal mechanisms for periodic review. Legislatures may include review clauses in genome-editing statutes or in broader biomedical legislation. Agencies may be required to publish periodic assessments of technological developments and their regulatory implications. Advisory bodies may be empowered to recommend updates to classifications, safety guidance, or evidentiary standards. The precise design will vary by jurisdiction, but the institutional principle is the same: regulatory review should be part of ordinary governance rather than an *ad hoc* response to failure.

Such review must have consequences. A periodic report that does not connect to guidance revision, classification change, enforcement priorities, or legislative reconsideration risks becoming symbolic. Technology-responsive review should therefore include a pathway from scientific development to regulatory action. For example, evidence that base editing produces distinctive bystander or off-target effects should trigger reconsideration of safety endpoints, assay requirements, and long-term monitoring standards rather than being treated as general scientific background.

### 6.2 Differentiated legal categorization of genome editing modalities and uses

TRR also requires more refined legal categorization. Existing systems often rely heavily on the distinction between somatic and germline editing. That distinction remains essential, but it is not sufficient. Genome editing now includes nuclease-based editing, base editing, prime editing, epigenome editing, multiplex editing, and diverse delivery platforms. These modalities differ in mechanism, risk profile, reversibility, clinical maturity, and evidentiary requirements.

A technology-responsive framework should therefore combine broad legal categories with modality-sensitive assessment. At the highest level, law may continue to distinguish basic research, preclinical research, somatic clinical use, embryo research, and reproductive or heritable use. Within those categories, however, regulators should account for the editing system, delivery method, target tissue, expected durability of effect, and available safety evidence. *Ex vivo* somatic editing in hematopoietic stem cells should not automatically be treated like *in vivo* systemic editing. Base editing should not be assessed solely through standards developed for double-strand-break-dependent CRISPR-Cas9. Prime editing may require different evidentiary attention again.

This does not mean that law should create endless micro-categories. Overly technical legal classification can become unstable and difficult to administer. The more realistic goal is to create a tiered framework that preserves administrability while requiring regulators to explain why particular technologies and uses are grouped together. Such explanation is important for legal accountability. It prevents both under-regulation, where new modalities evade scrutiny, and over-generalization, where heterogeneous technologies are treated as if they pose identical risks.

### 6.3 Scientific expertise within legal authority

Genome editing governance cannot function without scientific expertise. Regulators need technical advice to understand whether new editing systems alter safety assumptions, whether existing detection methods remain adequate, and whether new clinical pathways require different forms of monitoring. Yet expertise should not displace public authority. One danger in fast-moving biotechnology governance is that practical decision-making shifts into expert committees, professional guidance, or informal advisory processes whose legal status is unclear.

TRR therefore requires scientific-institutional integration. Expert advisory structures should be permanent enough to support continuity, interdisciplinary enough to capture the relevant scientific and ethical issues, and transparent enough to remain publicly accountable. They should include expertise in molecular genetics, clinical medicine, reproductive medicine, bioinformatics, toxicology, bioethics, law, and, where relevant, patient and community perspectives. Their role should be to identify regulatory significance, not to govern in place of law.

Agencies and legislatures should also be clear about how expert advice is used. Where expert committees recommend new safety endpoints, revised trial requirements, or reclassification of an editing modality, the responsible public body should explain whether and how those recommendations are adopted. This preserves a necessary distinction: science informs regulatory judgment, but it does not itself supply legal authority.

### 6.4 Institutional ethics review and its limits

Institutional ethics committees remain important in genome editing governance. They assess protocol-level risk, informed consent, participant protection, scientific justification, and local compliance. Their proximity to research practice gives them an important role that legislatures and national agencies cannot fully replace.

However, ethics review cannot carry the entire governance burden. The CRISPR twin incident showed that local review may fail when broader legal and institutional structures are weak. Ethics committees are often designed to assess individual protocols, not to settle system-wide questions about the legal permissibility of reproductive genome editing, embryo implantation, or boundary-crossing clinical uses. Their authority, expertise, independence, and enforcement capacity may also vary significantly across institutions.

TRR therefore calls for a more clearly delimited role for ethics committees. They should remain central to protocol-level review, but their work should be connected to higher-level regulatory pathways. High-risk genome editing proposals should trigger escalation to specialized national or regional review bodies. Ethics committees should also be supported by genome-editing-specific criteria, including attention to off-target detection, bystander edits, delivery risks, long-term follow-up, reproductive implications, data transparency, and justification for the selected editing modality. Ethics review should become more technically informed without becoming a substitute for public law.

### 6.5 Specialized oversight for high-risk applications

Some genome editing proposals raise issues that exceed ordinary institutional review. These include reproductive or heritable uses, embryo-related research with plausible translational implications, first-in-human *in vivo* editing with novel delivery systems, and interventions where uncertainty is unusually high or consequences may be irreversible. Such cases require specialized oversight.

Specialized oversight may take different forms. A jurisdiction might establish a national genome editing review body, a mandatory referral mechanism, an inter-agency committee, or a higher-level authorization process. The key point is not the creation of a new bureaucracy for its own sake. It is to ensure that unusually sensitive proposals are assessed consistently and transparently by institutions with appropriate authority and expertise.

This is especially important for preventing forum shopping. If high-risk proposals can be reviewed only by local institutional committees, researchers may seek permissive or weakly resourced review environments. A centralized or coordinated review mechanism can reduce this risk and clarify which decisions require public authority rather than institutional discretion alone.

### 6.6 Digital regulatory infrastructures

TRR also requires operational infrastructure. Static statutes and conventional review committees are not enough for a technology that develops through dispersed research networks and rapidly changing technical knowledge. Digital regulatory infrastructures can support visibility, coordination, and learning.

Three forms are particularly relevant. First, registry infrastructure can improve transparency by recording genome editing research, clinical trials, and high-risk proposals. Second, reporting infrastructure can standardize information about approvals, safety assessments, adverse events, monitoring obligations, and long-term follow-up. Third, horizon-scanning infrastructure can help regulators identify emerging modalities, delivery systems, safety concerns, and translational trends before they produce governance failure.

These tools should support legal judgment rather than replace it. Their purpose is not to automate regulatory decisions, but to improve the information environment in which those decisions are made. They are also important for international coordination. Shared or interoperable registries can make it harder for high-risk research to disappear into jurisdictional gaps and can support earlier regulatory learning across borders.

### 6.7 International coordination without assuming uniformity

Genome editing is transnational, but enforceable authority remains mostly national. This creates a persistent governance tension. Scientific collaboration, commercial development, clinical trial recruitment, and publication networks cross borders, while legal systems remain jurisdictionally bounded. A purely domestic approach is therefore incomplete, but a fully harmonized global legal regime is unlikely.

Three forms of coordination are especially important. Informational coordination can be supported through shared registries, common reporting expectations, and cross-border awareness of ongoing research and clinical activity. Normative coordination can promote convergence around baseline principles and prohibited or highly restricted practices, even where national law differs in detail. Regulatory coordination can support structured exchange among national agencies concerning evidentiary standards, safety signals, emerging modalities, and enforcement concerns.

TRR should aim for coordination without pretending that uniformity is immediately achievable. The WHO framework provides an important foundation, particularly in its emphasis on registries, transparency, ethical oversight, and international cooperation (World Health Organization, 2021a; World Health Organization, 2021b). A technology-responsive approach would connect such soft-law guidance more directly to domestic governance, including national review cycles, agency guidance, ethics committee criteria, and public reporting systems. Conversely, domestic regulatory experience should inform international learning. This two-way movement is more realistic than treaty-based harmonization and more robust than purely aspirational soft law.

### 6.8 Collaborative governance beyond regulators and scientists

Genome editing governance should not be a regulator-scientist dyad. Patient groups, disability advocates, reproductive medicine professionals, research institutions, funders, publishers, civil society organizations, and affected communities all shape the conditions under which genome editing develops and is judged legitimate (Jasanoff et al., 2019; Subica, 2023). Their roles differ, but each can identify concerns that may not be visible from within regulatory or scientific institutions alone.

Different stakeholders illuminate different forms of risk and legitimacy (Waltz et al., 2024; Scheinerman, 2023; Knight, 2026; Nelson et al., 2024). Patient groups may highlight therapeutic urgency and the cost of regulatory delay. Disability advocates may surface concerns about stigma, social meaning, and the risk that genetic intervention will reinforce narrow ideas of normality. Reproductive medicine professionals may identify practical risks in embryo-related pathways. Funders and journals can influence transparency, registration, reporting, and compliance by making these expectations conditions of support or publication. Civil society organizations can challenge overly narrow expert framings and bring broader social values into view.

Collaborative governance should not be romanticized. More actors can also mean more conflict, delay, and institutional complexity. But in a field where the legitimacy of regulation depends on more than technical safety, a governance architecture that excludes affected perspectives will be incomplete. TRR therefore treats collaborative governance as a source of both knowledge and public justification.

### 6.9 Enforcement and credibility

Adaptive governance is sometimes criticized for emphasizing learning while saying too little about enforcement (Cosens et al., 2017; Eshuis and Gerrits, 2021). That criticism is important for genome editing. A governance system that can revise guidance but cannot respond to deliberate violation will not sustain public trust. The lesson of the CRISPR twin incident is that norms must be backed by credible institutional consequences.

TRR therefore requires graduated but meaningful enforcement. Minor failures of reporting or documentation may justify correction, supervision, or temporary suspension. More serious breaches, such as unauthorized clinical use, concealment of high-risk experimentation, or violation of explicit prohibitions, should trigger stronger sanctions, including professional discipline, withdrawal of approval, institutional penalties, or criminal liability where appropriate. Enforcement should be proportionate, but it must be credible. Strong enforcement also helps distinguish responsible innovation from opportunistic experimentation. In genome editing, visible sanctions for serious violations serve not only deterrence, but also public reassurance that adaptive governance is not permissive drift.

### 6.10 Public engagement as part of regulatory design

Public engagement is often invoked in genome editing governance, but it is frequently left underdeveloped. In a TRR framework, engagement should not be treated as a rhetorical supplement to expert regulation or as a public communication exercise after major decisions have already been made. Genome editing raises questions about social meaning, distributive justice, reproductive responsibility, disability perspectives, and public authority over human biotechnology. These questions cannot be resolved by technical expertise alone.

A first requirement is to distinguish engagement from one-way communication. Public understanding of science remains important, but engagement should not be reduced to explaining genome editing to lay publics in order to secure acceptance. Many genome-editing controversies involve “wicked” questions for which there is no single technical answer, especially where applications affect human reproduction, environmental systems, disability communities, or access to medicine (Wirz et al., 2020). Engagement should therefore allow publics and stakeholders to question purposes, assumptions, priorities, and acceptable trade-offs.

A second requirement is to connect engagement to decision points. The decision-phases framework developed for gene editing in the wild distinguishes research and development, regulatory review, and deployment and management as stages at which different publics, stakeholders, and forms of input may be relevant (Barnhill-Dilling et al., 2021). Although developed for environmental biotechnology, this logic is also useful for biomedical genome editing. Early engagement can test assumptions about desirable uses and social priorities; engagement during regulatory review can inform standards for acceptable risk, consent, access, and oversight; and later engagement can support monitoring, public learning, and reassessment.

A third requirement is to avoid treating “the public” as a single body with stable preferences waiting to be recorded. Public engagement exercises can unintentionally produce the opinions they claim merely to capture, especially when they are designed around institutional needs for legitimacy rather than open deliberation (Breuer and Penkler, 2024). Engagement design should therefore be attentive to who participates, how questions are framed, what information is provided, who interprets the results, and whether public input has a visible pathway into regulatory revision.

Finally, engagement should be evaluated rather than assumed to be valuable by definition. Public participation may aim to improve decisions, satisfy democratic norms, redistribute voice, build civic capacity, or identify social concerns, but these are different objectives and require different evaluative criteria (Reynolds et al., 2023; Selin et al., 2017). TRR therefore links engagement to regulatory significance: the stronger a decision’s implications for future persons, affected communities, public trust, or the boundaries of acceptable use, the stronger the case for structured public and stakeholder involvement. In this sense, engagement is not merely an ethical aspiration. It is one way a technology-responsive legal system remains publicly accountable while it adapts. This also supports a more anticipatory engagement architecture, rather than waiting for public controversy to emerge around a specific application (Burall, 2018).

## 7 Discussion

The argument developed in this review reframes genome editing governance as a problem of regulatory design rather than only a debate over ethical permissibility. Questions about germline editing, embryo research, therapeutic risk, and reproductive use remain central, but they do not exhaust the governance challenge. Genome editing is difficult to regulate because the technology itself and social meanings continue to change. A governance framework that treats genome editing as a stable object risks becoming misaligned with the field it seeks to govern.

TRR responds to this problem by treating technological evolution as a legally relevant condition. Its aim is not to make law move at the same speed as science. That would be unrealistic and, in many contexts, undesirable. Law should not simply mirror scientific momentum or defer to technical possibility. Rather, TRR asks how legal institutions can remain capable of revision while preserving legality, transparency, accountability, and public justification. This is the central distinction between regulatory flexibility and legally structured adaptiveness.

**FIGURE 1:**
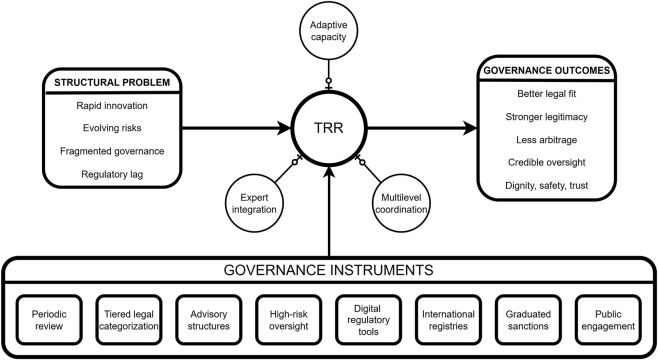
Conceptual model of technology-responsive regulation (TRR) for genome editing.

Figure 1 summarizes this logic. Scientific evolution produces pressure on existing legal categories and oversight practices. That pressure is not automatically translated into legal change. It must be interpreted through institutions capable of scientific assessment, legal reasoning, public engagement, and coordinated decision-making. TRR therefore links three core pillars—adaptive regulatory capacity, scientific-institutional integration, and multilevel coordination—to concrete governance instruments, including periodic review, differentiated categorization, specialized oversight, digital regulatory infrastructure, international coordination, enforcement, and structured public engagement. The model is best understood as a feedback architecture rather than a linear pathway. Regulatory decisions shape the conditions for research and clinical translation, while new scientific and social developments in turn create reasons for reassessment.

This approach has three implications for genome editing law. First, it challenges the assumption that stronger rules alone will solve governance failure. Strong rules are necessary in some contexts, especially where reproductive or heritable uses are concerned. However, rules that cannot be revised or interpreted in light of technological development may become either overbroad or under-protective. The problem is not only whether law is strict enough, but whether it remains fitted to the technology it governs.

Second, TRR clarifies why technical improvement does not eliminate governance concerns. Base editing and prime editing may reduce some risks associated with double-strand-break-dependent editing, but they also create different evidentiary questions. A more precise technology is not automatically a less governable or more governable one; it is a different regulatory object. This is why TRR emphasizes modality-sensitive assessment and dynamic evidentiary standards rather than a simple movement from prohibition to permission as science improves.

Third, TRR helps distinguish public accountability from expert management. Genome editing governance requires scientific expertise, but expert knowledge cannot by itself decide questions about acceptable risk, reproductive boundaries, justice, access, or the social meaning of genetic intervention. Public engagement, legal authority, and transparent reason-giving are therefore not external additions to technical regulation. They are part of what makes adaptive governance legitimate.

The framework also helps explain why existing governance arrangements are simultaneously important and incomplete. International soft-law reports, WHO recommendations, national regulatory pathways, ethics committees, and professional norms have all contributed to genome editing governance. They should not be dismissed as ineffective simply because they are fragmented. The more precise point is that they have not yet been organized into a sufficiently coherent system for continuous regulatory learning. TRR does not reject these instruments. It asks how they can be connected so that scientific developments, ethical concerns, and legal revisions move through identifiable and accountable pathways.

The model has relevance beyond genome editing. Similar governance problems appear in artificial intelligence, synthetic biology, neurotechnology, and data-driven health systems, where legal categories are repeatedly challenged by rapid technical change (Pantanowitz et al., 2024; Sun et al., 2022; Rainey, 2024; Niemiec et al., 2025). Genome editing is especially instructive because it combines scientific dynamism with unusually high moral stakes. It therefore offers a demanding test case for whether law can become adaptive without becoming unstable, technocratic, or merely reactive.

Several limits should be acknowledged. First, TRR is not a jurisdiction-specific legislative blueprint. Legal systems differ in constitutional structure, regulatory capacity, administrative tradition, and ethical commitments. A country with centralized biomedical governance may operationalize TRR differently from a federal system with fragmented authority. Second, TRR does not remove disagreement over substantive boundaries. Jurisdictions may reasonably differ on the legal status of embryo research, germline intervention, and reproductive use. The contribution of TRR is not to settle all such disagreements, but to provide a framework for governing them through more accountable and adaptive institutions. Third, this review focuses primarily on legal and regulatory design. Genome editing governance is also shaped by political economy, intellectual property, commercial incentives, research funding, publication norms, and global inequalities in scientific capacity (Borg and Policante, 2025). These forces influence which technologies are developed, which diseases receive attention, who gains access to resulting therapies, and whose values shape public debate. A complete account of genome editing governance must therefore connect regulatory design with these broader structures.

Even with these limits, the core claim remains: responsible genome editing governance requires more than stronger ethical principles or more frequent policy statements. It requires institutions capable of revising classifications, updating evidentiary standards, coordinating across levels of authority, and justifying decisions publicly as the technology changes. TRR is offered as a framework for that task.

## 8 Conclusion

Genome editing exposes a governance problem that is broader than the permissibility of any single application. This review has proposed TRR as a framework for addressing that problem. Its contribution is to organize familiar mechanisms—periodic review, scientific advice, differentiated categorization, registries, public engagement, enforcement, and international coordination—around a specific legal task: maintaining accountable regulation when the object of governance is scientifically dynamic. For genome editing, this means that law should not merely draw boundaries at a single point in time. A technology-responsive approach therefore seeks a middle path between rigid prohibition and open-ended flexibility. It asks legal systems to adapt without drifting, to learn without deferring entirely to expertise, and to support legitimate innovation without losing public accountability.
